# Impact of COVID-19 Social Distancing Mandates on Gastrointestinal Pathogen Positivity: Secondary Data Analysis

**DOI:** 10.2196/34757

**Published:** 2022-08-24

**Authors:** Tanner Palmer, L Scott Benson, Christina Porucznik, Lisa H Gren

**Affiliations:** 1 Division of Public Health Department of Family and Preventive Medicine University of Utah School of Medicine Salt Lake City, UT United States; 2 bioMérieux, Inc Salt Lake City, UT United States

**Keywords:** social distancing, gastrointestinal, COVID-19, gastroenteritis, surveillance, epidemiology, pathogen transmission, respiratory pathogen, public health, pathogen outbreak, outbreak, surveillance tool, diagnostic database

## Abstract

**Background:**

Acute gastrointestinal (GI) illnesses are of the most common problems evaluated by physicians and some of the most preventable. There is evidence of GI pathogen transmission when people are in close contact. The COVID-19 pandemic led to the sudden implementation of widespread social distancing measures in the United States. There is strong evidence that social distancing measures impact the spread of SARS-CoV-2, and a growing body of research indicates that these measures also decrease the transmission of other respiratory pathogens.

**Objective:**

This study aims to investigate the impact of COVID-19 social distancing mandates on the GI pathogen positivity rates.

**Methods:**

Deidentified GI Panel polymerase chain reaction test results from a routinely collected diagnostic database from January 1, 2019, through August 31, 2020, were analyzed for the GI pathogen positivity percentage. An interrupted time series analysis was performed, using social distancing mandate issue dates as the intervention date. The following 3 target organisms were chosen for the final analysis to represent different primary transmission routes: adenovirus F40 and 41, norovirus GI and GII, and *Escherichia coli* O157.

**Results:**

In total, 84,223 test results from 9 states were included in the final data set. With the exception of *E coli* O157 in Kansas, Michigan, and Nebraska, we observed an immediate decrease in positivity percentage during the week of social distancing mandates for all other targets and states. Norovirus GI and GII showed the most notable drop in positivity, whereas *E coli* O157 appeared to be least impacted by social distancing mandates. Although we acknowledge the analysis has a multiple testing problem, the majority of our significant results showed significance even below the .01 level.

**Conclusions:**

This study aimed to investigate the impact of social distancing mandates for COVID-19 on GI pathogen positivity, and we discovered that social distancing measures in fact decreased GI pathogen positivity initially. The use of similar measures may prove useful in GI pathogen outbreaks. The use of a unique diagnostic database in this study exhibits the potential for its use as a public health surveillance tool.

## Introduction

Acute gastrointestinal (GI) illnesses are of the most common problems evaluated by physicians, and they are also some of the most preventable [1]. There is evidence of GI pathogen transmission when people are in close contact, such as at mass gatherings and in group childcare [2-4]. Common infection control measures such as handwashing and limiting contact with sick individuals can lead to a decrease in GI pathogen transmission and illness [1]. Still, the United States alone sees 311 to 375 million episodes of acute GI illnesses per year, leading to more than 900,000 hospitalizations and 6000 deaths [1]. These illnesses are not only a burden to health systems, but they can also be incredibly uncomfortable, and in some instances, dangerous for the patient.

The COVID-19 pandemic led to the sudden implementation of widespread public health measures in the United States, including 6-feet social distancing protocols, messaging around effective hand hygiene, stay-at-home orders, large gathering bans, and the closures of public areas (eg, schools, restaurants, and nonessential businesses) [5-7]. There is strong evidence that social distancing measures impact the spread of SARS-CoV-2, and a growing body of research indicates that these measures also considerably decreased the transmission of influenza, respiratory syncytial virus, and respiratory enterovirus [8-13]. There was a reduction of reportable GI illnesses observed during the COVID-19 pandemic in countries that implemented public health measures, and data from the National Outbreak Reporting System showed a reduction in GI illnesses in the United States from 2019 to 2020 [14-16]. With this study, we hope to add to the growing knowledge base that public health measures meant to control COVID-19 also impacted other diseases.

Identification of GI pathogens has routinely relied on contemporary diagnostic microbiology; however, many laboratories are adopting rapid polymerase chain reaction (PCR) tests for the identification of GI pathogens from stool samples [17]. Adoption of rapid PCR tests, such as the BioFire FilmArray GI Panel (referred to as GI Panel), in conjunction with participation in automated diagnostic databases, like BioFire Syndromic Trends (referred to as Trend), allows for the investigation and monitoring of GI pathogen positivity rates at the level of communities or states [18].

Using Trend, this study aims to investigate the impact of COVID-19 social distancing mandates on the GI pathogen positivity rates in different states. Understanding the impact of the mandates on GI pathogens may allow for the expanded utility of these measures to control pathogens in the future. We hypothesize that social distancing measures meant to limit the transmission of COVID-19 also resulted in decreased GI pathogen positivity.

## Methods

### Ethical Considerations

This study was reviewed by the University of Utah Institutional Review Board (IRB) and was determined not to meet the definitions of Human Subjects Research according to Federal regulations (IRB_00142577). Therefore, the study did not require IRB oversight.

### Data Source and Collection

#### Test Results

To determine changes in positivity rates of pathogens, deidentified test results from the GI Panel from January 1, 2019, through August 31, 2020, were analyzed. The GI Panel is a widely deployed rapid PCR test, typically used in the hospital setting, designed to detect the most common pathogens associated with gastroenteritis [19]. To test on the GI panel, stool specimens are collected in Cary Blair transport medium from patients with gastroenteritis, and they are tested for 22 targets including bacteria, viruses, and parasites [19]. Three target organisms were chosen for this study: adenovirus F40 and 41, norovirus GI and GII, and *Escherichia coli* O157. These targets were selected because they represent different primary transmission routes. Adenovirus (types F40 and 41) is transmitted via aerosolized droplets and has a high prevalence in children. Norovirus (GI and GII) is mainly transmitted through the fecal-oral route and is highly contagious. *E coli* O157, although rare, is a cause of foodborne bacterial illnesses, and because of the severity of the disease, it is likely to be tested for and detected when it occurs [1,20].

Deidentified test results including the date of the test, the target organism species, the number of positive tests for that target, and the total number of tests were automatically recorded in the Trend database [18]. All participating laboratories were hospital- or clinic-based and accredited by the Clinical Laboratory Improvement Amendments. Reference laboratories were excluded. Laboratory verification or quality control runs were automatically excluded from Trend. Data used in this study were only from laboratories in the United States. For the purposes of data deidentification, 3 laboratories must participate in Trend in a state for that state to be included in the database. As such, 9 states were included in this study: California, Colorado, Illinois, Kansas, Michigan, Nebraska, Ohio, Texas, and Wisconsin.

#### Social Distancing Mandates

The start dates of individual state COVID-19 social distancing mandates were obtained from the State COVID-19 Data and Policy Actions data curated by the Kaiser Family Foundation [21]. The following 4 key mandates were chosen to be included in this analysis: (1) stay-at-home orders, (2) restaurant closures, (3) nonessential business closures, and (4) large gathering bans.

Mask mandates were not included in the analysis.

### Statistical Analysis

Data analysis was performed using Stata/IC (version 16.1; StataCorp). Daily test results were provided in the Trend database. Days when no GI Panel tests were performed in a state were excluded from the analysis. The daily test results were summed to weekly test results, and subsequently, weekly positivity percentages for each state and target were analyzed.

**Figure 1:**
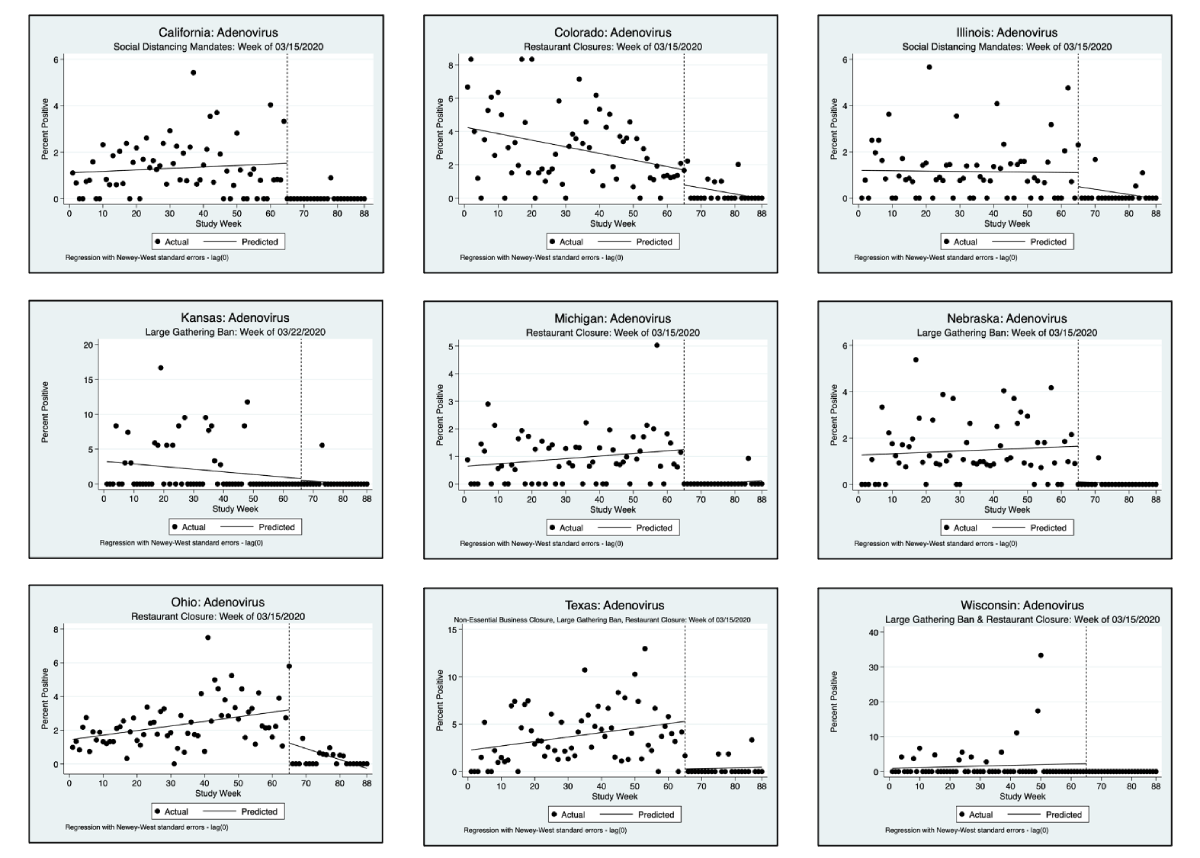
Interrupted time series analysis (ITSA) graphs for adenovirus F40 and 41. With the exception of California and Illinois, a second ITSA was performed for the second week in which social distancing mandates were issued. Refer to Figure 2 for the second ITSA.

**Figure 2:**
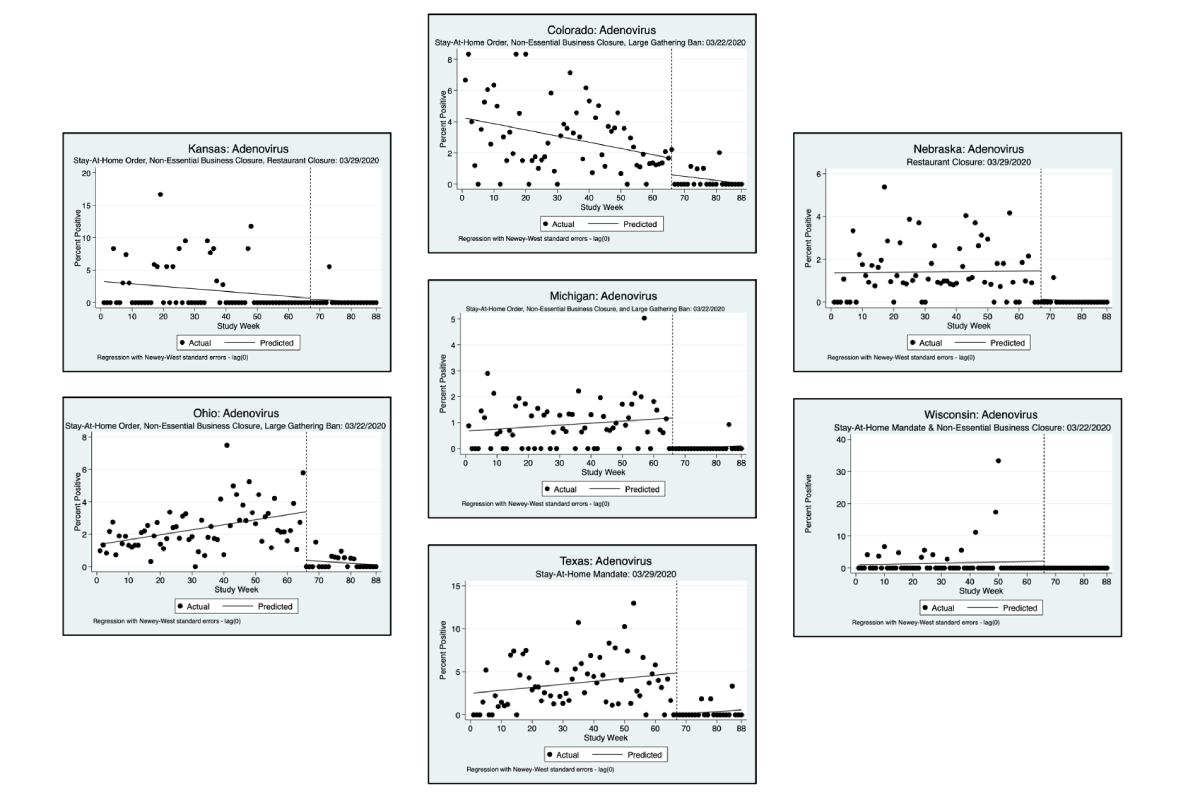
Interrupted time series analysis (ITSA) graphs for adenovirus F40 and 41 for the second week in which social distancing mandates were issued.

**Figure 3:**
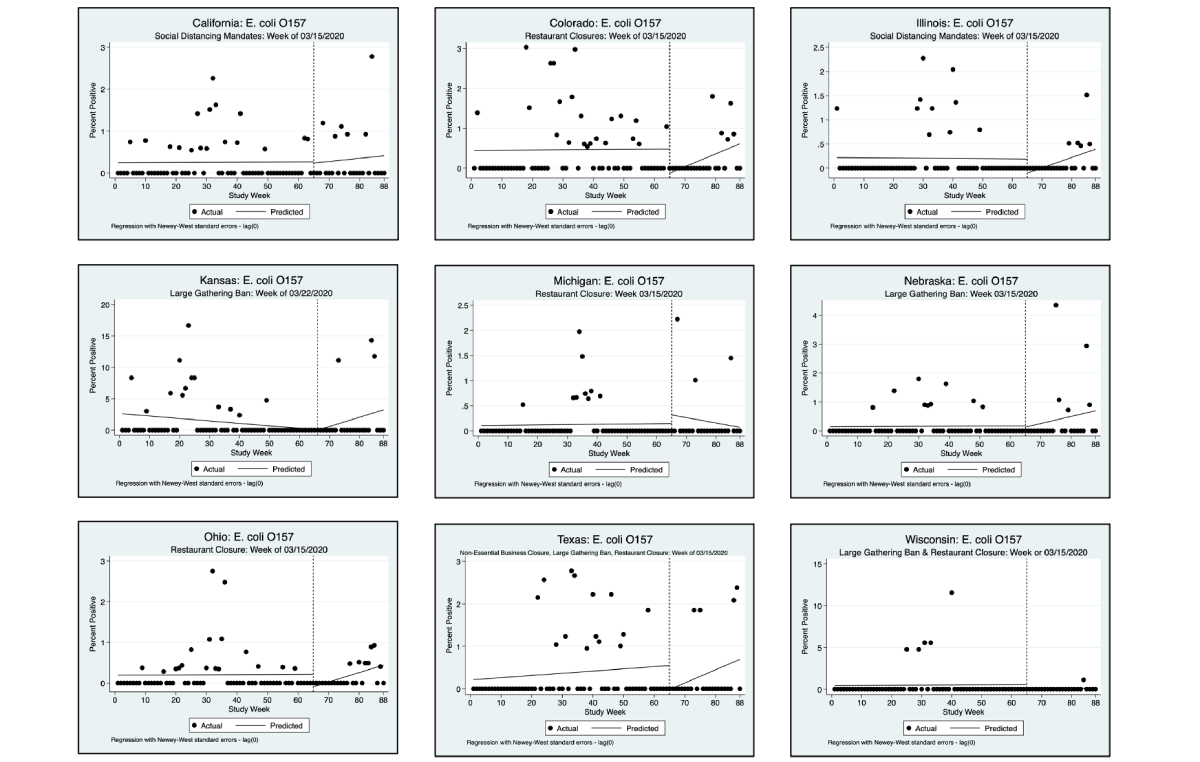
Interrupted time series analysis (ITSA) graphs for *Escherichia coli* O157. With the exception of California and Illinois, a second ITSA was performed for the second week in which social distancing mandates were issued. Refer to Figure 4 for the second ITSA.

**Figure 4:**
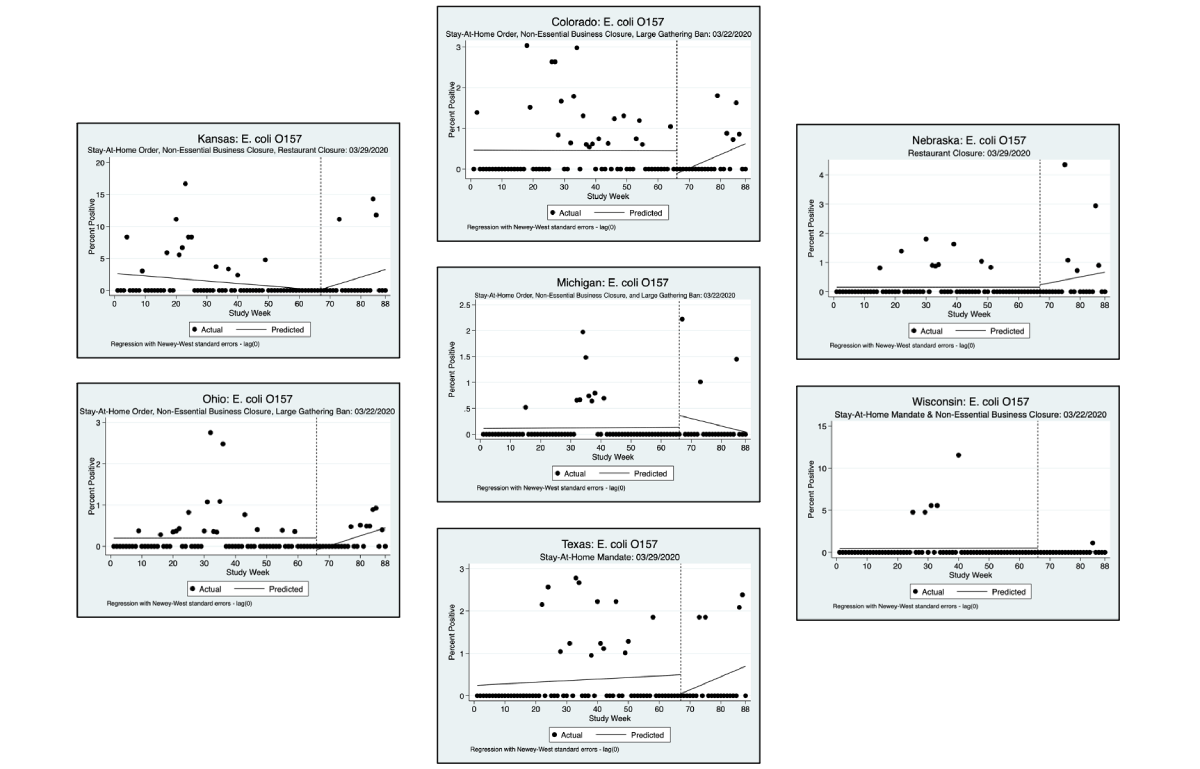
Interrupted time series analysis (ITSA) graphs for *Escherichia coli* O157 for the second week in which social distancing mandates were issued.

**Figure 5:**
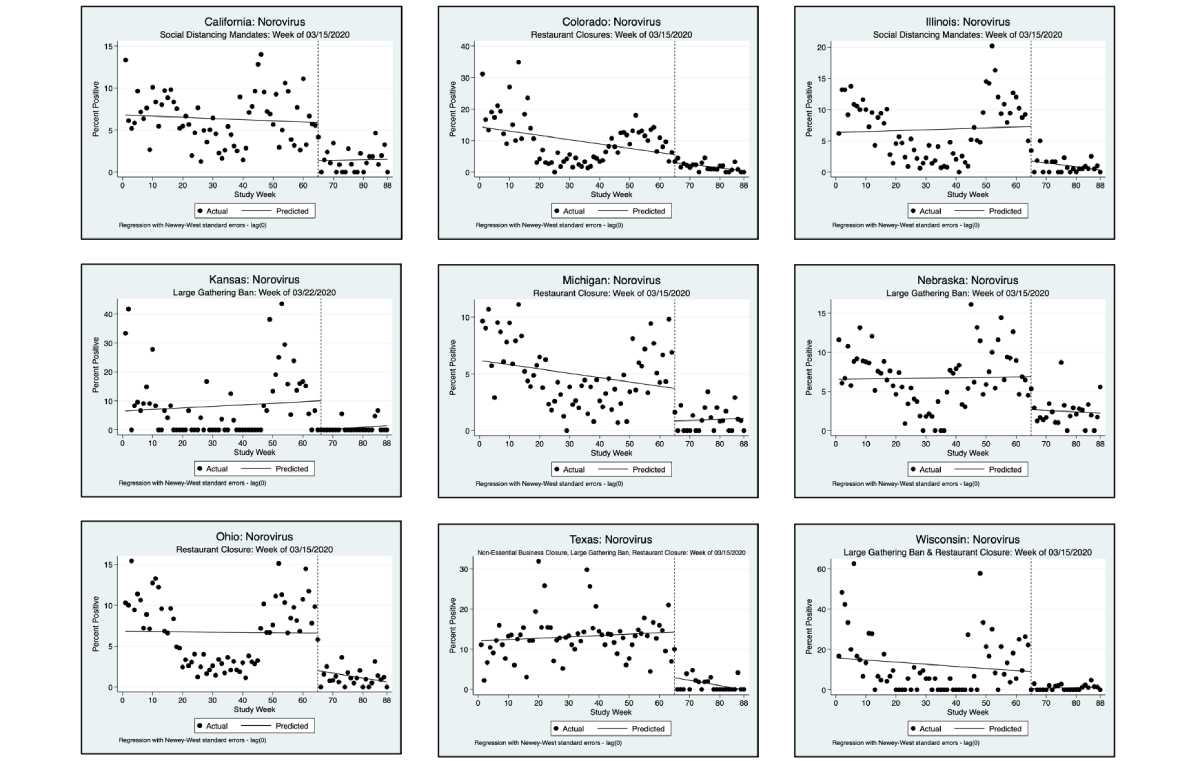
Interrupted time series analysis (ITSA) graphs for norovirus GI and GII. With the exception of California and Illinois, a second ITSA was performed for the second week in which social distancing mandates were issued. Refer to Figure 6 for the second ITSA.

**Figure 6:**
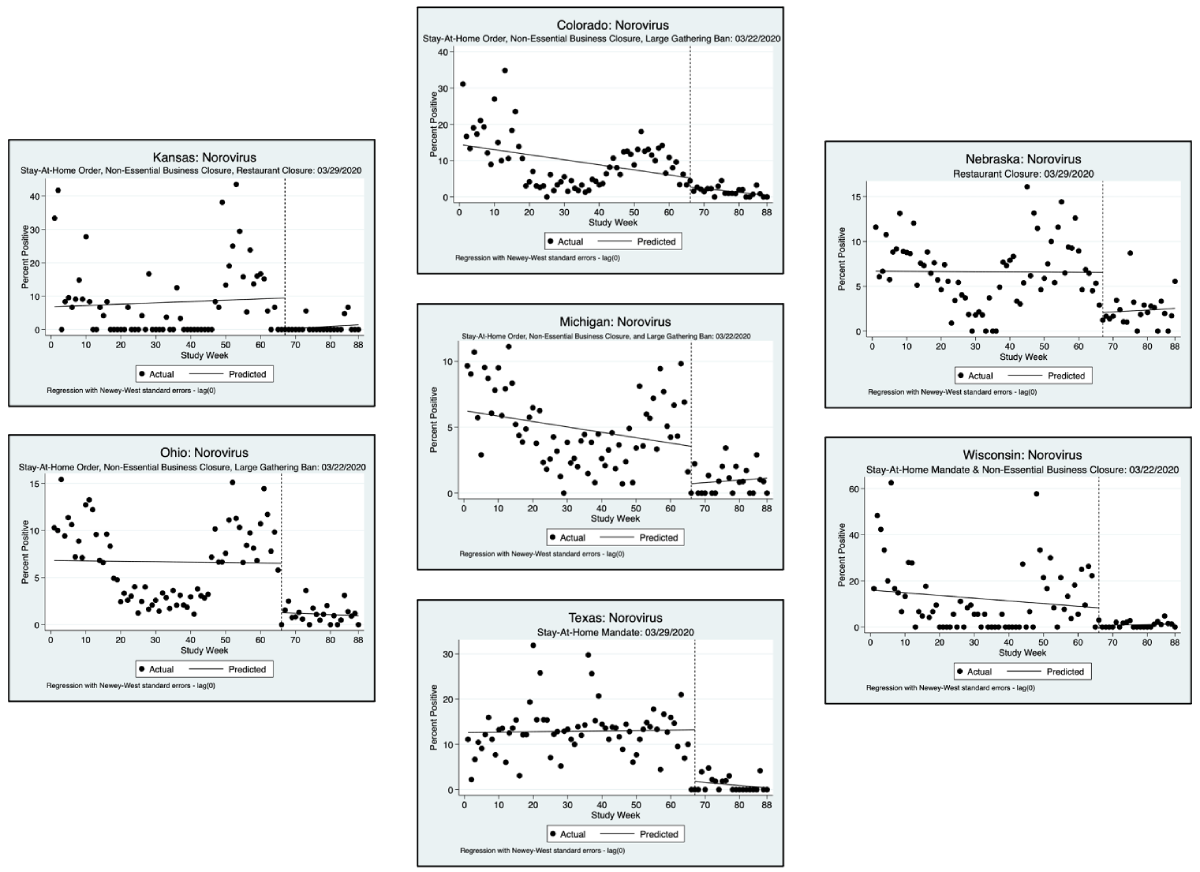
Interrupted time series analysis (ITSA) graphs for Norovirus GI and GII for the second week in which social distancing mandates were issued.

An interrupted time series analysis (ITSA) was performed for each of the 3 pathogens of interest and each state, using a downloadable Stata package (Figures 1-6) [22]. Figures 1-6 show the percent positivity before social distancing mandates went into effect (solid black dots before the vertical dashed line) and after social distancing mandates went into effect (solid black dots after the vertical dashed line). The best fit positivity percentage is represented by the solid horizontal line. We were most interested in the difference between the best fit positivity percentage before social distancing mandates and immediately after social distancing mandates. The time of intervention for the ITSA was the week that social distancing mandates went into effect for each individual state. In most states, multiple mandates went into effect in the same week; thus, the ITSA was performed only once for mandates occurring in the same week. Mandates occurring in separate weeks required a separate ITSA.

## Results

### Data Overview

A total of 84,223 tests from 9 states were included in the final data set (Table 1). Norovirus GI and GII had a higher overall positivity percentage compared to adenovirus F40 and 41 and *E coli* O157.

All states included in the analyses mandated social distancing policies in March of 2020 between the weeks beginning March 15, 2020, and March 29, 2020 (Table 2). Social distancing mandates occurred across two separate weeks for most states, with the exception of California and Illinois, where all mandates were announced in a single week. According to the Kaiser Family Foundation, Nebraska was the only state in the analysis that did not mandate nonessential business closures or a stay-at-home order.

**Table 1:** Distribution of total tests and positive tests.

States	Total tests, n	Positive tests		
		Adenovirus F40 and 41, n (%)	Norovirus GI and GII, n (%)	*Escherichia coli* O157, n (%)
California	11,400	124 (1.09)	614 (5.39)	32 (0.28)
Colorado	9879	222 (2.25)	693 (7.01)	45 (0.46)
Illinois	11,268	104 (0.92)	594 (5.27)	25 (0.22)
Kansas	1674	26 (1.55)	109 (6.51)	24 (1.43)
Michigan	11,458	88 (0.77)	486 (4.24)	16 (0.14)
Nebraska	8814	97 (1.10)	489 (5.55)	20 (0.23)
Ohio	21,049	404 (1.92)	1171 (5.56)	45 (0.21)
Texas	6103	195 (3.20)	685 (11.22)	24 (0.39)
Wisconsin	2588	20 (0.77)	173 (6.68)	8 (0.31)

**Table 2:** Timing of social distancing mandates in analyzed states.

State	Week of mandate			
	Stay-at-home order	Restaurant closures	Nonessential business closures	Large gathering bans
California	3/15/20	3/15/20	3/15/20	3/15/20
Colorado	3/22/20	3/15/20	3/22/20	3/22/20
Illinois	3/15/20	3/15/20	3/15/20	3/15/20
Kansas	3/29/20	3/29/20	3/29/20	3/22/20
Michigan	3/22/20	3/15/20	3/22/20	3/22/20
Nebraska	N/A^a^	3/29/20	N/A	3/15/20
Ohio	3/22/20	3/15/20	3/22/20	3/22/20
Texas	3/29/20	3/15/20	3/15/20	3/15/20
Wisconsin	3/22/20	3/15/20	3/22/20	3/15/20

^a^N/A: not applicable; according to the Kaiser Family Foundation, Nebraska did not mandate nonessential business closures or a stay-at-home order.

### Immediate Effect

**Figure 7:**
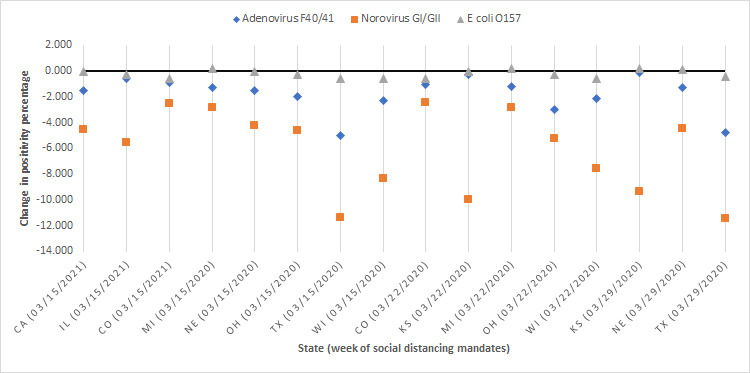
Immediate change in positivity percentage during the week of social distancing mandates.

With the exception of *E coli* O157 in Kansas, Michigan, and Nebraska, we observed an immediate decrease in positivity percentage during the week of social distancing mandates. Norovirus GI and GII showed the most notable drop in positivity, whereas *E coli* O157 appeared to be least impacted by social distancing mandates (Figure 7; Table 3). Although we acknowledge the analysis has a multiple testing problem, the majority of our significant results showed significance even below the .01 level (Table 3).

**Table 3:** Immediate change in positivity percentage during the week of social distancing mandate issuance.

State (week of mandate)				Adenovirus F40 and 41				Norovirus GI and GII				*Escherichia coli* O157		
				Change (%)		*P* value		Change (%)		*P* value		Change (%)		*P* value
		California (3/15/20)	–1.499		<.001		–4.507		<.001		–0.027		.90	
		Illinois (3/15/20)	–0.607		.22		–5.527		<.001		–0.290		.02	
		Colorado (3/15/20)	–0.901		.08		–2.560		.08		–0.601		.001	
		Michigan (3/15/20)	–1.278		<.001		–2.870		.001		0.171		.58	
		Nebraska (3/15/20)	–1.536		<.001		–4.195		.001		–0.042		.89	
		Ohio (3/15/20)	–1.963		.03		–4.583		.001		–0.307		.004	
		Texas (3/15/20)	–5.016		<.001		–11.347		<.001		–0.564		.049	
		Wisconsin (3/15/20)	–2.279		.16		–8.364		.03		–0.582		.10	
		Colorado (3/22/20)	–1.048		.04		–2.471		.09		–0.561		.001	
		Kansas (3/22/20)	–0.283		.73		–9.986		.003		–0.075		.95	
		Michigan (3/22/20)	–1.210		<.001		–2.827		.001		0.224		.50	
		Ohio (3/22/20)	–3.009		<.001		–5.252		<.001		–0.287		.006	
		Wisconsin (3/22/20)	–2.159		.16		–7.569		.046		–0.549		.11	
		Kansas (3/29/20)	–0.133		.87		–9.361		.004		0.228		.93	
		Nebraska (3/29/20)	–1.320		<.001		–4.475		<.001		0.082		.82	
		Texas (3/29/20)	–4.777		<.001		–11.428		<.001		–0.447		.15	

## Discussion

### Principal Findings

The results from this study indicate that public health measures meant for COVID-19 can initially decrease GI pathogen positivity. In most instances, we observed an immediate decrease in positivity percentage, suggesting that social distancing measures can very quickly decrease GI pathogen transmission. Similar results have been observed in previous studies, demonstrating that these public health measures can decrease transmission of other pathogens transmitted through the respiratory route, such as influenza, respiratory syncytial virus, and respiratory enterovirus; this study additionally demonstrates that they are also effective against GI pathogens on a national scale [8-13].

To provide context for our results on the effects of the mode of transmission of the pathogens, we chose 3 pathogens with different routes of transmission. Interestingly, our results show norovirus GI and GII was most impacted by the social distancing mandates. This may be due to the contagious nature of the pathogen, suggesting that social distancing mandates may be most effective against highly contagious GI pathogens that are most commonly spread person to person. The reduced person-to-person contact resulting from social distancing mandates could have decreased the transmission of norovirus GI and GII. Conversely, social distancing mandates showed a minimal impact on *E coli* O157 positivity rates. The results of *E coli* O157 positivity may be due to the incubation period of the bacterial infection, typically longer than viral infections, as we did not account for lag time in our analysis; however, this could be investigated in future studies. Additionally, as restaurants closed to dine-in service, take-out service typically remained available, which could have contributed to the minimal impact observed in our results. The social distancing mandates exhibited a moderate effect on the positivity percentage of adenovirus F40 and 41, more so than on *E coli* O157, but not as extreme as on norovirus GI and GII. It is possible that limited person-to-person contact impacted one route of transmission for adenovirus F40 and 41, but the virus was still spreading through other routes of transmission (eg, a fecal-oral route) leading to the observed moderate impact.

Our results show variability in the change of positivity percentage between states. This study did not aim to analyze this variability; however, differences in health behaviors of state residents and the enforcement of social distancing mandates may be contributing causes of this variability. An area of focus for future studies could be investigating differences between states.

The most notable strength of this study is the database itself, Trend. This study is a novel use of this unique diagnostic database that allowed us access to a large sample size of routinely collected, deidentified data. The large sample size consisting of test results across the nation should allow for generalizability to many communities. During the COVID-19 pandemic, social distancing mandates were universally used as a public health measure to control transmission, permitting us to use them as a variable in the analysis and compare states. The ITSA analysis allowed us to investigate the immediate impact of COVID-19–related social distancing measures on the positivity percentage of GI pathogens.

Although this study presents interesting findings using a unique diagnostic database, it is not without limitations. The BioFire Trend database is expansive, and not all laboratories in a state may be participating, introducing the possibility of selection bias. However, the data set represents data from 9 states in different regions across the United States, allowing for some generalizability. There is possibility for diagnostic bias if a clinician chooses not to use the GI Panel, but most provider institutions will have testing algorithms established, that likely include the GI Panel if the patient is showing acute GI illness symptoms. Additionally, key social distancing measures were only measured on a statewide basis, which does not account for differences between counties or cities, nor did we have available data about compliance with social distancing mandates. However, given the time frame of our study, compliance is likely high, since our analysis period covers the time when the only measures available to prevent COVID-19 were social distancing measures. Hand hygiene was not investigated in this study, and it may have impacted the transmission of GI pathogens during the COVID-19 pandemic; future studies should investigate the effect of hand hygiene. BioFire Trend does not collect demographic data, and further studies are needed to investigate the effects of gender, age, race or ethnicity, and other demographic variables on GI pathogen positivity percentages.

### Conclusions

We investigated the impact of social distancing mandates for COVID-19 on GI pathogen positivity, and we discovered that social distancing measures did, in fact, decrease GI pathogen positivity. Our results show the possible utility of social distancing measures to reduce the spread of GI pathogens. The use of similar measures may prove useful in GI pathogen outbreaks. In addition to anecdotal evidence of decreased illness during the COVID-19 pandemic, the findings from this study can be used to reinforce that social distancing interventions can be used to reduce GI pathogen transmission.

The use of a unique diagnostic database, Trend, exhibits the potential for its use as a public health surveillance tool. We have only demonstrated one use of this routinely collected data, but we imagine it could be used for algorithms, models, and tools for early detection of diseases and monitoring the impact of different interventions to control outbreaks. Further research should not only investigate additional applications of Trend but also the impact of different public health measures between different communities. Further studies could assess whether GI pathogen positivity also decreased on a global scale during the COVID-19 time frame, further investigate the impact of COVID-19 mandates on pathogen positivity with the addition of contextual information, and observe long-term GI pathogen positivity after social distancing mandates went into effect.

## Abbreviations

GI: gastrointestinal
IRB: institutional review board
ITSA: interrupted time series analysis
PCR: polymerase chain reaction
